# Strategies for Promoting Doula Inclusivity in the Labor and Delivery Setting

**DOI:** 10.1007/s10995-025-04051-4

**Published:** 2025-02-20

**Authors:** Holly DeBernard Perkins, Christine Isaacs

**Affiliations:** 1 VCU Health Labor and Delivery Main Hospital, 6th Floor 1250 E Marshall Street, 23298 Richmond, VA USA; 2 Department of Obstetrics and Gynecology, VCU Health, Richmond, VA USA

**Keywords:** Doula, Obstetric Labor, Natural Childbirth, Labor Coach

## Abstract

**Purpose:**

Our facility aimed to establish a standardized process to guide healthcare team members to incorporate doulas in the labor and delivery setting while meeting regulatory and safety requirements and promoting operational transparency.

**Description:**

Doulas provide emotional, physical, and educational support to patients and families throughout the pregnancy, birth, and postpartum journey. Doula care has been identified with improved maternal and neonatal birth outcomes, as well as improved perceptions of the birthing process.

**Assessment:**

Our facility lacked a standard operating procedure for successfully incorporating doulas into the healthcare team.

**Conclusion:**

We developed strategies to promote doula inclusivity in our labor and delivery environment with safety and quality at the core of our focus.

## Significance

Doulas are trained to provide continuous labor support to positively impact childbirth outcomes. Establishing resources to integrate doulas in the labor and delivery setting contributes to improved birth outcomes, patient satisfaction, and the underlying goal of patient safety with the provision of high quality, patient-centered birth experiences. Engaging doulas into the healthcare team requires a culture of acceptance, an operational plan, clear role delineation, and recognition of the benefits that a doula provides. The steps and process developed at Virginia Commonwealth University Medical Center (VCUMC) have proven to be a low-cost way to promote doula inclusivity in the labor and delivery environment.

## Introduction

Doulas are a valuable part of the pregnancy journey and help support the patient and birth family during their prenatal, birth, and postpartum experience. Doulas are trained childbirth professionals who support and supplement the clinical staff to provide a high quality, patient-centered birth experience, having formed a well-established relationship and “birth plan” with the patient prior to the beginning of labor; doulas complement the prenatal education and health care team (McLeish & Redshaw, 2018). Traditionally, patients hire and compensate doulas outside of their primary obstetric provider.

Doulas can positively impact outcomes for women and infants, including increased chances of spontaneous vaginal birth, decreased rates of Cesarean or operative delivery, and shorter duration of labor (Bohren et al., 2017). Lower rates of any analgesia, including epidurals, are noted. Doula support can also contribute to lower rates of preterm birth (Kozhimannil et al., 2016) and increased rates of breastfeeding initiation (Thurston et al., 2019; Hans et al., 2018).

A doula’s continuous labor support improves the quality of the patient’s childbirth experience without any noted evidence of associated harm. They can increase or improve patient confidence, satisfaction, overall quality of care, health literacy, and communication between patients and providers (Akhavan & Lundgren, 2012; Thurston et al., 2019; Hans et al., 2018). Their continuous physical presence empowers patients through periods of rapid change and growth.

With the goal of integrating doulas into our L&D health care team, clear role delineation and hospital employee acceptance of a doula’s work were essential, while keeping the vision of patient safety and provision of a high quality, patient-centered birth experience.

## Purpose

Doulas have been attending births at Virginia Commonwealth University Medical Center (VCUMC) for several decades. The COVID-19 pandemic highlighted our need to limit visitor access but conflicted with promoting doula presence. Initial doula monitoring efforts were arduous, timely, and lacked a standard operating procedure (SOP). We recognized that doulas’ longstanding commitment to patient experience was not being met with equal appreciation.

Notably, our facility anticipated additional doula presence with the introduction of the Virginia Department of Medical Assistance Services Community Doula Program that offers doula services to Medicaid members.

Our initiatives aimed to streamline the process to promote doula engagement, inclusivity, and successfully assimilate doulas (that were not employed by the hospital) with the interdisciplinary healthcare team. This paper describes the innovations accomplished at VCUMC to meet the regulatory requirements, safety, and operational transparency necessary to engage doulas in the labor environment with a patient-centered and staff-supported model, despite many inherent obstacles.

## Description

### Site Description

VCUMC is an urban academic medical center located in Richmond, Virginia that performs over 3,000 births per year. VCUMC is a tertiary care referral hospital providing high-risk obstetrical services with access to a Level IV Neonatal Intensive Care Unit. Despite the full spectrum of obstetrical services provided and the complex nature of our patient referrals, VCUMC maintains a Cesarean delivery rate below 30%. As a teaching institution, our patient-centered multidisciplinary teams include obstetric anesthesiologists, anesthesia residents, certified registered nurse anesthetists, obstetricians, OB/GYN residents, registered nurses (RNs), and CNMs. Notably, VCUMC holds a Society of Obstetric Anesthesia and Perinatology Center of Excellence designation.

### Rationale for Change

L&D units are traditionally locked for infant security; it is imperative to closely monitor individuals who are authorized to enter and exit the unit. Our efforts to promote doula support were developed during the COVID-19 public health emergency, which was a time focused on social distancing and *minimizing* visitors in the environment. We were met with conflicting goals: promoting doula engagement while still ensuring infant security and patient safety with COVID-19 disease transmission concerns. Additionally, the Virginia Department of Medical Assistance Services launched the Community Doula Program in 2022, which increases access for Medicaid patients by providing doula services as a benefit (Virginia Department of Medical Assistance Services, 2025). Without this service, Medicaid patients would need to hire and pay for doulas out of pocket.

With increased state funding, more doulas would be entering the environment. VCUMC wanted to embrace the services doulas offer while safely incorporating them into the L&D atmosphere. Beginning in 2022, we began developing our SOP and identified several gaps:

1. We had no clear doula role delineation to serve as a blueprint for safe operations;
2. We had no system for doula training verification;
3. We had no written protocol to guide VCUMC team members as to how to identify and engage doulas with appropriate boundaries for patient safety and transparency;
4. We did not have a dedicated space for doulas to rest while promoting continuous labor support.

The gaps were used as a model to reach our goal of promoting doula inclusivity and required a multifaceted, interdisciplinary approach. The processes listed herein were conducted in accordance with prevailing ethical principles and did not require institutional review board approval prior to implementation.

### Doula Education and Scope

While doulas’ practices may differ, they are typically on-call, likely involved in the early labor process, and accompany patients to the hospital (Young, 2022). While in the birthing facility, a doula provides patient-centered support but does not administer medical care or preform interventions.

Doula training is not standardized, lending to some of the confusion when a doula presents to support a patient in labor but has varying levels of training and experience. There are multiple organizations that train and provide doula certification (Young, 2022) and one study suggests that a doula with even a modest amount of training is beneficial to the birthing person (Bohren et al., 2017). Volunteer doula programs can provide meaningful continuous labor support to a greater number of patients (Kivlighan et al., 2022), and therefore, VCUMC welcomes all doulas into the environment.

Virginia Medicaid patients can connect with a doula through the Department of Medical Assistance Services (Virginia Department of Medical Assistance Services, 2025). However, if doulas choose to certify with a non-state affiliated organization, such as DONA International, directories connected with that organization can help patients connect with a doula (DONA International, 2025).

As of January 2022, the Virginia Department of Health established minimum requirements for state-certified doulas and provided certification through the Virginia Certification Board (Virginia Department of Medical Assistance Services, 2025). In Virginia, any doula may attend a birth (Virginia Certification Board, 2022), but for eligible Virginians to utilize Medicaid services, they must utilize a state-certified doula (Virginia Department of Medical Assistance Services, 2025). There are currently 112 state-certified doulas in Virginia with many other doulas not recognized by the state.

### Assessment

To create buy-in and obtain feedback, multiple disciplines were engaged to facilitate safe guidelines that would allow for a doula’s presence in the labor room (see Table 1). Adequate preparation for change and creation of an organizational plan will result in cultural changes required to implement interventions (Vaughn et al., 2019).

**Table 1:** Steps to promote Doula Inclusivity in Labor and Delivery at VCU Medical Center

Task	Solution
1. Establish a culture and buy-in from physician and nursing teams	Hold multidisciplinary workgroups to discuss gaps in the Standard Operating Procedure (SOP), how to symbiotically include doulas in the inpatient setting, how to hardwire the process
2. Engage and collaborate with other departments to streamline processes for doula inclusivity	Write a hospital policy outlining parameters for doula inclusivity in the inpatient setting
3. Have an attestation for the parameters that a doula is engaged in	Create a QR code form where doulas check in to Labor and Delivery and attest to their training
4. Have a system that visually identifies the doula and thus, an understanding of their role	Create doula-specific identification sticker
5. Have ways to support the doula during their work	Create a dedicated doula respite room to promote rest and self-care
6. Incorporate all members of the team to provide safe, high quality birth experiences	Utilize the SOP for doula inclusivity in daily practice to promote patient safety and satisfaction

### Doula Training Attestation

The initial work began with the Risk Management and Safety and Security Departments, who needed to address the management of doula training and certifications. This posed several challenges since doulas are not VCUMC employees, making it difficult to audit and manage the certification documentation. Initial options considered were a paper file with doula certifications or a signed paper consent, which would greatly increase the paper and storage burden on unit clerical team members.

Furthermore, while a doula is considered a visitor in the hospital setting, their presence goes beyond what the visitor policy should govern. VCUMC also wanted to open the option to student doulas who were working towards their certification.

An efficient process was created where a doula submits their information upon entry to the L&D unit when they present to support a patient. They scan a QR code poster, which opens an online form to enter their name and affiliated doula organization (or if they independently practice). It also requires an attestation to having doula certification or training and to abide by hospital policies, which are individually created for doulas and visitors.

The information from this form can be exported to a spreadsheet with a date and time stamp of the doula’s entry, allowing for automated tracking. The Risk Management and Safety and Security Departments advised that this process would be acceptable while reducing the burden on the front-line team.

### Doula Policy

To engage doulas in the labor unit in a safe, equitable, and inclusive manner, a formal hospital policy was needed. Collaboration between Risk Management, Safety and Security, the obstetric care providers, the obstetric anesthesia team, and nursing leadership provided valuable input. The policy defines what a doula is, criteria for entry into the unit, and their scope of practice in the hospital setting.

Direct doula participation in the policy drafting was strongly considered but not performed due to information sharing requirements previously established at our facility. We paid close attention to strategies that elevate a doula’s presence in our environment while remaining within the established hospital guidelines. We took individual feedback from doulas and incorporated the changes through our interdisciplinary policy review process.

The importance of including parameters for a doula’s presence in the operating room (OR) was thoughtfully detailed. While our traditional practice is to allow only one support person in the OR, the team considered the additional benefit that doulas provide to patients who require a Cesarean delivery. If clinically appropriate, our policy allows the team to decide if a doula may be present in the OR with the caveat to make this determination prior to transferring the patient into the surgical area.

**Fig. 1:**
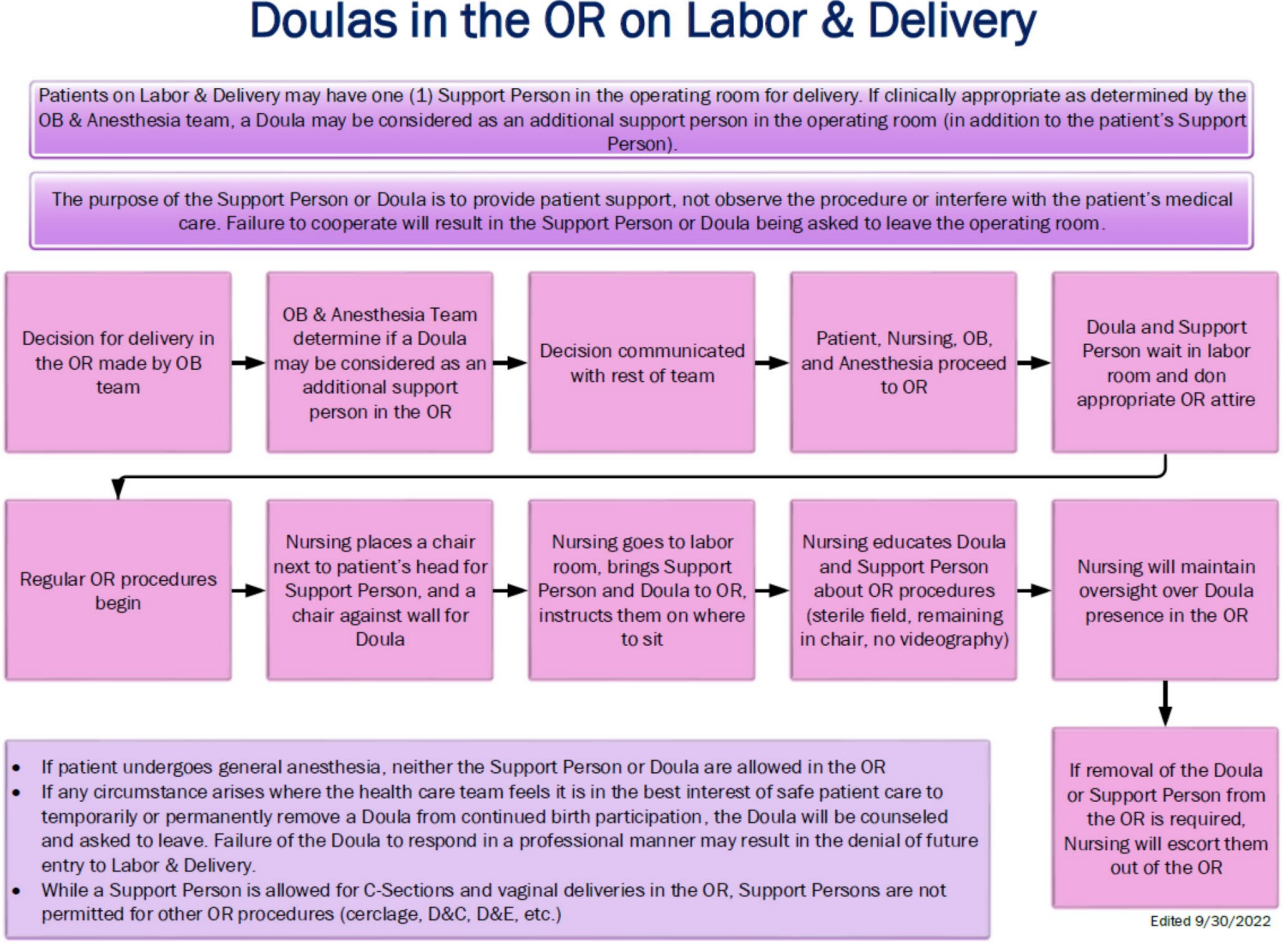
Process map for doula presence in the labor and delivery operating room

Nursing would be responsible for the doula in the OR and to ensure that the doula’s presence did not interfere with safe surgery. A process map was created to clearly delineate the roles in the OR (see Fig. 1). A doula would be provided a designated chair to sit in the OR until the birth of the baby; then, they would be permitted to ambulate between the patient, their partner, and the baby while promoting breastfeeding and assisting with skin-to-skin care. The specific placement of the doula and spatial arrangements are for patient safety and to allow the team to effectively manage the patient’s clinical needs.

### Identification of Doulas in the Environment

A doula’s skills elevate them from the typical hospital visitor and as such, it was imperative to clearly identify doulas to our health care teams. At VCUMC, visitors must wear a designated “VISITOR” sticker with the patient’s room number, the date, and location. We created a similar process for identifying doulas. After completing the online form via QR code, doulas receive a confirmation screen and present this confirmation to the registration clerk. They then receive a sticker with the word “DOULA” in bold, but also identifies them as a visitor. The doula’s name, patient’s room number, unit, and date are also visible; no protected health information is shown on the sticker. These efforts ensure clear role delineation between a patient’s visitor, doula, and members of the VCUMC team in the environment.

### Creating a Dedicated Space for Doula Respite

**Fig. 2:**
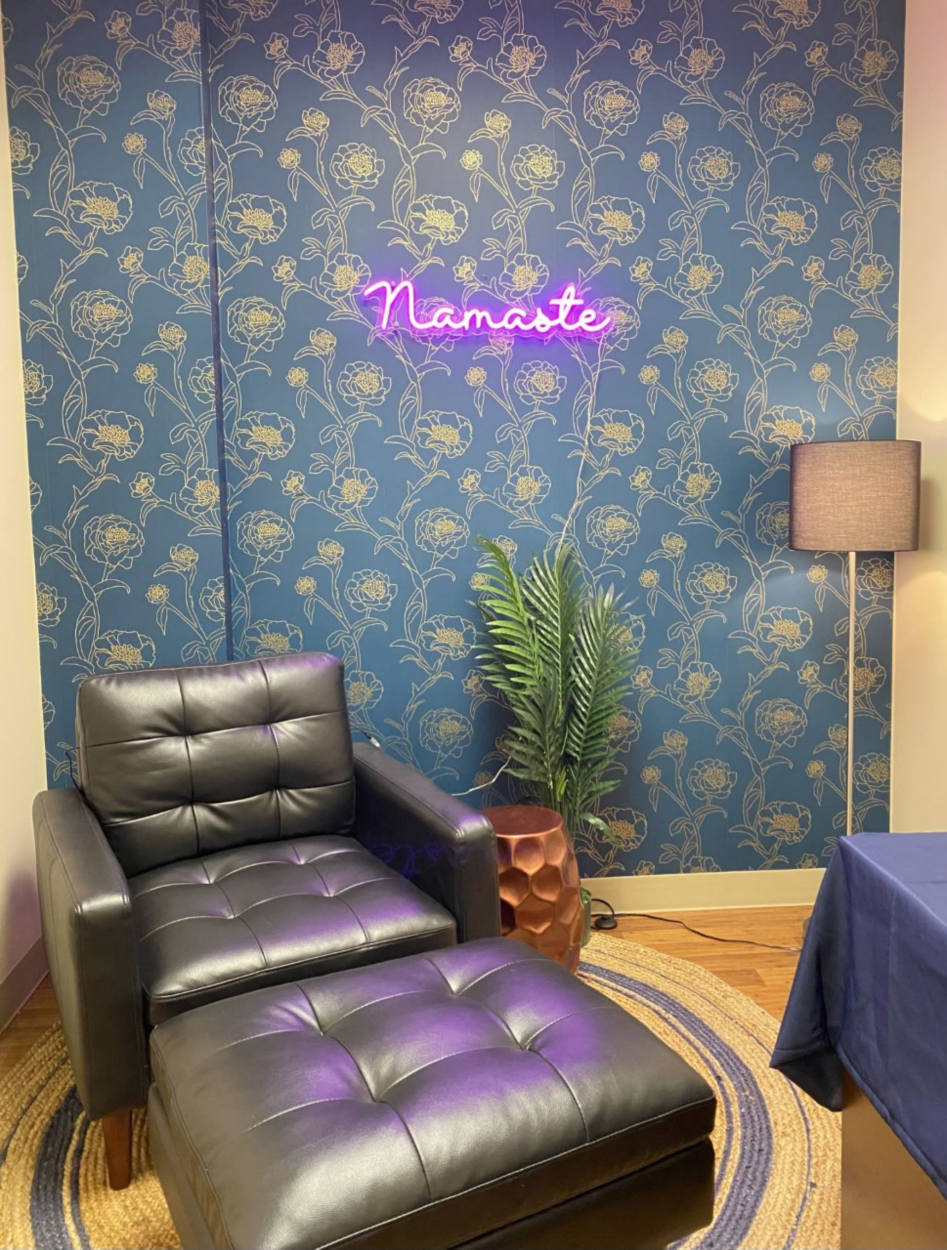
Doula respite room

To garner further inclusivity for doulas and to highlight the influential work that they do, a doula respite room was created. This is a designated space on L&D where the doula can go to rest and recharge, and then return to their patient to provide the best care possible (see Fig. 2). Ease of access to the room was paramount and as such, was stationed in a non-restricted area. An office was allocated and converted for this purpose.

The respite room design aimed to create a space that did not seem like it was inside the hospital. Low-level lighting was accomplished with a neon sign and a floor lamp. Removable wallpaper was hung, a rug was placed on the floor, and a comfortable chair and ottoman were ordered. Of note, a non-porous, wipeable fabric was chosen so that environmental services could wipe down all surfaces within the room after each use.

A designated table and small refrigerator were purchased. Food and Nutrition Service staff regularly stock refreshments to provide additional amenities to the room. A local artist donated artwork with a maternal theme to enhance the visual comfort of the space.

Access to the room is secured by a code-punch lock. This code is given to the doula after completing the online form and presenting the confirmation screen to the registration clerk.

The Plant Operations and the Strategy and Marketing Departments also created the signage for the room and designated the space as such on the facility map. Nursing and physician leadership advocated for the respite room to promote the patient satisfaction and safety principles associated with doula care; funding for the room was charged to the unit’s cost center.

Creating a doula respite room promotes continued inclusivity of our doulas and has built an atmosphere of trust and cultural acceptance. A doula’s work is demanding, and adjusting the environment in which they work reduces their burden of providing continuous labor support (Young, 2022). The room is a physical representation of the gratitude extended to the doula for providing support to our patients and communities by promoting high quality birth experiences.

### Education and Sustaining the Change

Attitudes surrounding doula care can impact the success of how well doulas are incorporated into the hospital care team (Neel et al., 2019). Lack of collaboration among team members may also prohibit successful implementation of new processes (Kivlighan et al., 2022). To appropriately integrate the new SOP, educational sessions were held during staff meetings, grand rounds, and leadership rounding.

Doula education was provided through a “Meet the Doula” event held at our hospital by our Strategy and Marketing Department. The event featured an interdisciplinary panel of healthcare team members that highlighted the new SOP and answered any questions from doulas in attendance. The event also offered a tour of our new doula respite room.

Promoting engagement from all levels of the healthcare team, including doulas, was important to ensure continued success with doula presence on the L&D unit. Since the implementation of our strategies, staff feedback has been overwhelmingly positive. There is a clearer understanding of expectations from every healthcare team member and a streamlined SOP to guide our facility.

## Conclusion

Doulas contribute to improved birth outcomes and patient satisfaction. Engaging doulas into the healthcare team requires a culture of acceptance, an operational plan, clear role delineation, and recognition of the benefits that a doula provides. Continuing that culture requires strong leadership and a commitment to interdisciplinary collaboration from all levels of the healthcare team.

The SOP developed at our institution has proven to be an easily attainable way to promote doula inclusivity in the L&D environment, without exaggerated oversight from the nursing management team. While future opportunities for collecting information on specific patient, visitor, doula, and team member satisfaction exist, the SOP ensures that our institution maintains regulatory compliance while promoting safe, high-quality care.

The strategies illustrated here assimilated doulas into the birthing journey at VCUMC. Creating a transparent process with a written policy provided guidance to our team members and created a positive understanding of doula support within the entire healthcare team.

## Data Availability

Not applicable.
